# IFN-Lambda (IFN-λ) Is Expressed in a Tissue-Dependent Fashion and Primarily Acts on Epithelial Cells In Vivo

**DOI:** 10.1371/journal.ppat.1000017

**Published:** 2008-03-14

**Authors:** Caroline Sommereyns, Sophie Paul, Peter Staeheli, Thomas Michiels

**Affiliations:** 1 Université catholique de Louvain, de Duve Institute, MIPA-VIRO 74-49, Brussels, Belgium; 2 Department of Virology, University of Freiburg, Freiburg, Germany; University of California Irvine, United States of America

## Abstract

Interferons (IFN) exert antiviral, immunomodulatory and cytostatic activities. IFN-α/β (type I IFN) and IFN-λ (type III IFN) bind distinct receptors, but regulate similar sets of genes and exhibit strikingly similar biological activities. We analyzed to what extent the IFN-α/β and IFN-λ systems overlap in vivo in terms of expression and response. We observed a certain degree of tissue specificity in the production of IFN-λ. In the brain, IFN-α/β was readily produced after infection with various RNA viruses, whereas expression of IFN-λ was low in this organ. In the liver, virus infection induced the expression of both IFN-α/β and IFN-λ genes. Plasmid electrotransfer-mediated in vivo expression of individual IFN genes allowed the tissue and cell specificities of the responses to systemic IFN-α/β and IFN-λ to be compared. The response to IFN-λ correlated with expression of the α subunit of the IFN-λ receptor (IL-28Rα). The IFN-λ response was prominent in the stomach, intestine and lungs, but very low in the central nervous system and spleen. At the cellular level, the response to IFN-λ in kidney and brain was restricted to epithelial cells. In contrast, the response to IFN-α/β was observed in various cell types in these organs, and was most prominent in endothelial cells. Thus, the IFN-λ system probably evolved to specifically protect epithelia. IFN-λ might contribute to the prevention of viral invasion through skin and mucosal surfaces.

## Author Summary

Virus-infected cells can secrete interferons (IFNs), cytokines that induce an infection-resistant state in neighboring cells. IFNs are critical to slow down early multiplication of pathogens in the body. Two IFN families exhibiting strikingly similar properties were described: type I IFNs (or IFN-α/β) and type III IFNs (or IFN-λ). Our work addressed the question of the redundancy of these two IFN systems in vivo. First, we found that the relative expression of IFN-λ over that of IFN-α/β exhibited some extent of tissue specificity and was low in the brain. Next, we used a strategy based on in vivo expression of cloned IFN genes to compare the responses of different tissues to IFN-α and IFN-λ. As was suggested by previous in vitro work, response to IFN-λ appeared to be restricted to epithelial cells, unlike response to IFN-α which occurred in most cell types. Tissues with a high epithelial content such as intestine, skin or lungs were the most responsive to IFN-λ and expressed the higher amounts of IFN-λ receptor. Our data suggest that the IFN-λ system evolved as a specific protection of epithelia and that it might contribute to prevent viral invasion through skin and mucosal surfaces.

## Introduction

Type I interferon (IFN), also called IFN-α/β, was originally discovered owing to its potent antiviral activity [1]. Type I IFN was later shown to display pleiotropic activities. It modulates innate and acquired immune responses, cell growth and apoptosis [2].

Type I IFN forms a vast multigenic family [3]. Human and mouse genomes carry 13 or 14 genes coding for closely related IFN-α subtypes [4],[5]. In addition, they contain genes coding for IFN-β, IFN-κ [6], IFN-ε/τ [7] and IFN-ω (human) or limitin/IFN-ζ (mouse) [8]. MuIFN-α subtypes share about 90% amino acid sequence identity with each other and approximately 30% sequence identity with other type I IFN subtypes. Some of these IFNs are glycosylated while others are not [4],[5],[9],[10]. In spite of this remarkable variability, all type I IFN subtypes appear to bind the same heterodimeric receptor [11], raising the question of the reason for type I IFN gene multiplicity. Some data suggest that various IFN subtypes might exhibit different affinities for each of the receptor subunits and hence, generate signals that could vary in nature, duration, or intensity. For instance, Jaitin and his collaborators reported that IFN-α/β subtypes differ in their affinity for IFNAR1 and that this receptor subunit is the limiting factor for ternary complex formation [12]. Binding to the IFNAR1 subunit would favor signaling pathways leading to antiproliferative activity whereas binding to the IFNAR2 subunit would favor signaling pathways leading to antiviral responses [13]. Such subtle binding differences could explain the few qualitative differences observed in the activity of different IFN subtypes. Alternatively, the multigenic nature of the IFN family could allow individual IFN subtypes to be expressed in a tissue or in a cell-specific fashion.

Intriguingly, the multigenic type I IFN system cohabits with the seemingly redundant type III IFN system discovered more recently. Type III IFN (also called IFN-λ or IL-28/29) is structurally and genetically close to the members of the IL-10 family of cytokines but displays type I IFN-like activity [14],[15]. In humans, 3 genes code for the 3 members of this new family: IFN-λ1, IFN-λ2 and IFN-λ3. Among these molecules, only HuIFN-λ1 is glycosylated [14],[15]. In the mouse, the IFN-λ1 gene is a pseudogene. IFN-λ2 and IFN-λ3 genes encode glycosylated proteins [16].

IFN-λ expression has been shown to depend on the same triggers (viral infection, TLR ligands) [17],[18] and signal transduction pathways [19]–[21] as those inducing type I IFN expression. Type I and type III IFNs bind unrelated heterodimeric receptors. The type I IFN receptor is made of the ubiquitously expressed IFNAR1 and IFNAR2c subunits [22]. The type III IFN receptor is made of the IL-10Rβ subunit which is widely expressed and shared by other IL-10 related cytokines, and of the IL-28Rα subunit which is specific to IFN-λ and responsible for signal transduction [14]–[16],[23]. Although type I and type III IFN receptors are unrelated, they trigger strikingly similar responses, mostly through the activation of STAT-1 and STAT-2, and, to a lesser extent, of STAT-3 [14], [16], [24]–[26]. Association of phosphorylated STAT-1 and -2 with IRF-9/p48 yields the ISGF3 complex which induces the transcription of hundreds of genes, the so-called “interferon stimulated genes” (ISGs). These ISGs encode proteins such as Mx1, OAS or IFIT, which mediate the antiviral effects of IFN [27]. IFN-α/β and IFN-λ were also reported to activate the MAP kinase pathway through JNK and p38 phosphorylation. ISGs activated by type I and type III IFNs were found to be similar [25],[26]. Accordingly, type III IFN was shown to display antiviral [23],[24],[28],[29], antiproliferative [16],[30], and immunomodulatory properties [31],[32], similar to those of type I IFN.

It has been shown that, in vitro, cell responses to IFN-λ closely depend on the expression of the IL-28Rα receptor subunit [18],[26]. Overexpression of IL-28Rα in non-responding cells restored the response of these cells to IFN-λ [26]. IL-28Rα expression has been detected in primary keratinocytes and colonic cells, but not in splenocytes, fibroblasts and endothelial cells, indicating that the IFN-λ receptor can be expressed in a cell-specific fashion [16],[24]. These data suggest that, in vivo, distinct cells or tissues might be targeted by IFN-α/β and IFN-λ. However, few data are available about production of IFN-λ and about the tissue and cell specificity of the response to this IFN in vivo.

To examine possible tissue specificity of IFN-λ expression, we compared the expression of type I and type III IFNs in the brain and in the liver, using various viral infection models. To compare the responsiveness of different tissues and cells to type I and type III IFNs, we used a strategy based on in vivo expression of cloned IFN genes. We observed some tissue specificity in the production of type III IFN and a clear tissue specificity in the response to type III IFN. At the cellular level, the response to IFN-λ showed a marked specificity for epithelial cells, thus clearly differing from the response to IFN-α.

## Results

### Tissue dependency of type III IFN gene expression

Currently available in vitro data do not reveal differential expression of type I and type III IFN genes. To test whether some tissue specificity exists in the production of type III versus type I IFN in vivo, we compared IFN-α, IFN-β and IFN-λ expression in the brain and in the liver of mice infected with various RNA viruses: Theiler's virus (TMEV, the neurovirulent strain GDVII or the persistent strain DA1), LACVdelNSs (La Crosse virus mutant lacking the IFN-antagonist protein NSs), Mouse Hepatitis virus (MHV, strain A59) or Lactate dehydrogenase-elevating virus (LDV).

**Figure 1:**
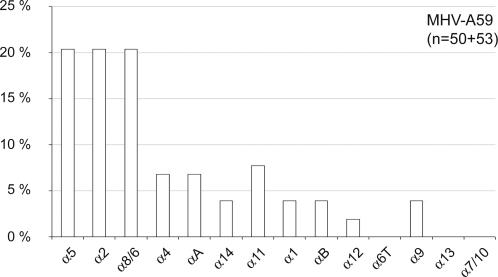
Relative expression of the various IFN-α subtypes in MHV-A59 infected livers. IFN-α coding sequences were amplified by RT-PCR using a primer mixture designed to amplify equally the different murine IFN-α subtypes [33]. PCR products were then cloned and individual clones were sequenced. The histogram shows the percentage of sequences from 2 mice (50 and 53 sequences) corresponding to each IFN-α subtype.

For detection of mouse IFN-λ, we designed new primers that amplify both IFN-λ2 and IFN-λ3 transcripts, but not putative transcripts from the IFN-λ1 pseudogene. For detection of mouse IFN-α, we designed primers that are specific for IFN-α5 (Table 1). This IFN subtype has been shown to be among the most prominently induced IFN-α subtypes in the brain, after both LACV and TMEV infections [33]. Using the RT-PCR-cloning-sequencing strategy used in the former study [33], we observed that IFN-α5 was also among the most prominently expressed IFN-α subtypes (20.4%) in the liver of MHV-infected mice (Figure 1). Thus, IFN-α5 expression appears to be a good marker to follow global IFN-α expression in both infected livers and brains.

**Table 1:** Primers sequences and PCR conditions used.

Gene amplified (Accession^a^)	Primer sequence^b^	Annealing temp. (°C)	Standard curve
β-actin (NM_007393.3)	(s) 5′- AGA GGG AAA TCG TGC GTG AC	60	genomic DNA or pTM793
	(as) 5′- CAA TAG TGA TGA CCT GGC CGT		
IFN-α5 (NM_010505.2)	(s) 5′- CCT GTG TGA TGC AAC AGG TC	62.5	pcDNA3-IFN-α5
	(as) 5′- TCA CTC CTC CTT GCT CAA TC		
IFN-β (NM_010510.1)	(s) 5′- ATG AAC AAC AGG TGG ATC CTC C	60	genomic DNA
	(as) 5′- AGG AGC TCC TGA CAT TTC CGA A		
IFN-λ2 (AY869695.1) and IFN-λ3 (AY869696.1)	(s) 5′- AGC TGC AGG CCT TCA AAA AG	64.4	pEF-IFN-λ2/-λ3
	(as) 5′- TGG GAG TGA ATG TGG CTC AG		
OASl2 (NM_011854.2)	(s) 5′- GGA TGC CTG GGA GAG AAT CG	60	pCS40
	(as) 5′- TCG CCT GCT CTT CGA AAC TG		
Mx1 (NM_010846.1)	(s) 5′- TCT GAG GAG AGC CAG ACG AT	60	pCS65
	(as) 5′- ACT CTG GTC CCC AAT GAC AG		
IFNAR1 (NM_010508.2)	(s) 5′- CAT GTG TGC TTC CCA CCA CT	60	pTM901
	(as) 5′- TGG AAT AGT TGC CCG AGT CC		
IL-28Rα (NM_174851.2)	(s) 5′- TGC AGA TTC CTC TCC AGC AA	60	pTM903
	(as) 5′- GTC TTC ACC CCC TGA AAC CA		

a Genbank accession number (http://www.ncbi.nlm.nih.gov/Genbank/).

b (s) sense primer; (as) antisense primer.

We first analyzed IFN production in mice infected intracerebrally (i.c.) with MHV-A59 or intraperitoneally (i.p.) with LDV (Table 2). Following i.c. injection, MHV-A59 can spread within the central nervous system (CNS), by the hematogenous and neuronal routes. The virus can also enter the bloodstream via the disrupted blood-brain-barrier at the inoculation site and reach the liver where it replicates. MHV-A59 strain is known to target a large range of cells including hepatocytes, macrophages (including Kupffer cells and microglial cells), endothelial cells, glial cells and neurons [34]. LDV injected i.p. rapidly infects a population of LDV-permissive macrophages in the mouse [35]. One day post-infection, which corresponds to the peak of viremia, LDV antigen-positive cells have been detected in most organs, including the liver and the leptomeninges of the brain. Subsequently, the virus establishes a persistent infection that is limited by the number of available target cells [36],[37]. Thus, groups of C57BL/6 mice were infected either i.c. with MHV-A59 or i.p. with LDV, since these infection models allow to compare the IFN responses in the brain and the liver of the same animals. Mice infected with MHV-A59 were sacrificed at 72h post infection, when clinical signs of encephalitis were prominent. LDV-infected mice were sacrificed at 24 hours post infection, which corresponds to the peak of viremia and of IFN expression [36],[38].

**Table 2:** Mouse infections with RNA viruses.

Virus	Route	Mouse strain	Mouse Age (weeks)	Infected Mice (n = )	Days p.i.	Organs	Figure
LDV	i.p.	C57BL/6	3–4	9	1^a^	Brain-Liver	2 A.
MHV-A59	i.c.	C57BL/6	3–4	10	3^b^	Brain-Liver	2 B.
TMEV-DA1	i.c.	129/Sv	3–4	2	5^c^	Brain	2 C.
LACVdelNSs^e^	i.p.	B6.A2G-Mx1	6	2	7^b^	Brain	2 D.
TMEV-GDVII^e^	i.c.	FVB/N	3–4	2	5^b^	Brain	2 E.
TMEV-GDVII	i.c.	C57BL/6	3–4	6	4^b^	Brain	2 F.
MHV-A59	i.p.	C57BL/6	3–4	7	2^d^	Liver	2 G.
MHV-A59	i.p.	129/Sv	5	3	2^d^	Liver	2 H.

a 1 day post-infection was reported to correspond to the peak of LDV replication and of IFN expression in vivo.

b Mice infected with highly neurovirulent viruses were sampled when signs of encephalitis were prominent (generally less than 24 hours before death).

c The DA1 strain of TMEV produces a transient encephalitis lasting about 1 or 2 weeks. In mice with the H-2 b haplotype, the virus is then rapidly cleared by the cytolytic T lymphocyte response [40],[41]. Mice were sampled at 5 days post-infection, a time-point representative of the acute phase of infection.

d Preliminary RT-PCR experiments failed to reveal a clear difference in IFN expression and viral load between 129/Sv mice infected i.p. for 2 days and for 7 days. Only mice with amounts of MHV-A59 detectable by conventional RT-PCR were taken into account.

e These samples from TMEV (GDVII strain) and LACVdelNSs infected brains were from a previous work [33].

**Figure 2:**
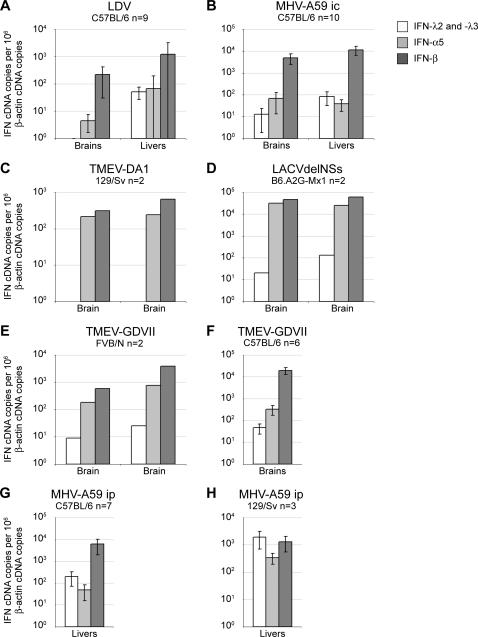
Quantification of IFN-λ, IFN-α5, and IFN-β transcripts in virus-infected brains and livers. Histograms show the number of IFN cDNA copies per 10^6^ β-actin cDNA copies, determined by real-time PCR, after reverse transcription of RNA extracted from the brain and liver of mice infected with different RNA viruses, in different experimental settings (see Table 2). A, B, F, G, H: mean and standard deviation of groups of mice. C–E: data from individual mice. Background amplification in mock-infected mice (not shown) was less than 1 copy of IFN-β or IFN-λ, and less than 10 copies of IFN-α5 cDNA, per 10^6^ copies of β-actin cDNA.

In mice infected with LDV (Figure 2A), we noticed a striking difference in the relative expression of IFN-λ in the brain and in the liver. IFN-λ mRNA was readily detected in the liver but was hardly detectable in the brain (1 out of 9 mice had detectable amounts of IFN-λ mRNA in the brain). In contrast, IFN-α and IFN-β mRNAs were clearly detected in both the liver and the brain of these mice. The expression of IFN-λ, relative to that of type I IFN was significantly lower in the brain than in the liver (Table 3). In mice infected with MHV-A59 (Figure 2B), the same trend was observed. The differences were less extensive, yet statistically significant (Table 3).

**Table 3:** Relative type III and type I IFN gene transcription in the brain and in the liver of infected C57BL/6 mice.

Mice	Virus (inoculation)	n	(IFNs compared)	Liver mean±SD^a^ type III/type I	Brain mean±SD^a^ type III/type I	p value^b^
C57BL/6	LDV (i.p.)	9	IFN-λ/IFN-α5	2.010±0.345	<0.284±0.096	0.0003
			IFN-λ/IFN-β	0.081±0.015	<0.008±0.004	0.0002
C57BL/6	MHV-A59 (i.c.)	10	IFN-λ/IFN-α5	2.703±0.541	0.294±0.118	0.0001
			IFN-λ/IFN-β	0.008±0.002	0.004±0.001	0.0147

a Calculations were as followed: for each mouse, the ratio between type III and type I IFN transcripts (IFN-λ/IFN-α or IFN-λ/IFN-β was calculated for the liver and for the brain. Mean ratios were then calculated for liver samples and for brain samples.

b Mann-Whitney p values testing whether the mean ratio between type III and type I expression measured in the liver differs significantly from that measured in the brain.

We then examined, in diverse experimental infection conditions (see Table 2), whether the same trend of lower relative expression of IFN-λ in the brain than in the liver existed. IFN production was examined in the brain of mice infected with neurotropic viruses (TMEV-DA1, TMEV-GDVII, LACVdelNSs). At the time point analyzed (Table 2), both TMEV strains inoculated intracerebrally primarily infect neurons, as do LACVdelNSs inoculated intraperitoneally [39]–[41]. In the brain of mice infected with these viruses, IFN-λ expression was either non-detectable (TMEV-DA1) or very low, compared to that of IFN-α or IFN-β (TMEV-GDVII and LACVdelNSs) (Figure 2C, 2D, 2E, 2F). In contrast, in the liver of mice infected i.p. with MHV-A59, although variation existed between experimental groups, IFN-λ expression was close to or higher than that of IFN-α (Figure 2G, 2H). Taken together, our data suggest that IFN-λ expression (relative to that of IFN-α/β) is restricted in the brain as compared to the liver.

### In vivo expression of IFN genes by electrotransfer

We next analyzed whether the response to specific IFNs also exhibited some degree of tissue specificity in vivo. To this end, we chose to compare ISGs expression in peripheral organs and in the CNS, after in vivo expression of cloned IFN genes. IFN was expressed in vivo from expression vectors that were electroinjected in the tibialis anterior muscle [42]. An advantage of this technique over the administration of recombinant IFN is that gene products are expected to carry native post-translational modifications like glycosylation.

**Figure 3:**
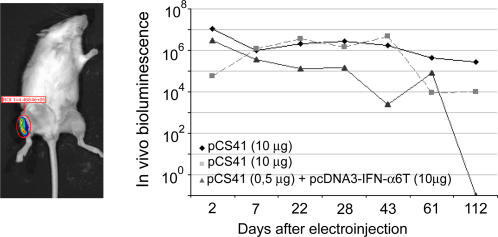
Expression of luciferase, in vivo, after i.m. electroinjection of an expression plasmid. Left panel: picture of a mouse showing luciferase expression in the tibialis muscle of the right leg. Right panel: follow-up of luciferase expression in vivo (arbitrary units), in two mice electroinjected with 10 µg of plasmid DNA (pCS41) expressing the firefly luciferase gene and in one mouse electroinjected with 0.5 µg of pCS41 plasmid DNA and 10 µg of plasmid DNA expressing IFN-α6T.

**Figure 4:**
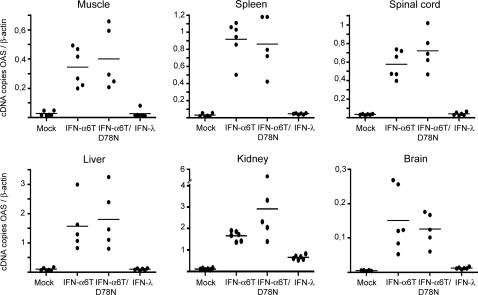
OASl2 expression in different tissues after electroinjection of plasmids coding for IFN-α or IFN-λ. OASl2 transcripts detected by real-time RT-PCR, 7 days after electroinjection of plasmid coding for MuIFN-α6T, MuIFN-α6T/D78N, MuIFN-λ3 or the empty vector (mock), in 7 week-old FVB/N mice. Results are expressed as OASl2 cDNA copies per β-actin cDNA copy. Graphs present results for individual mice and the mean for each group, for one representative experiment.

**Figure 5:**
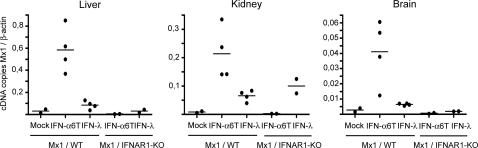
Mx1 gene expression induced by systemic IFN-α and IFN-λ, in organs of IFNAR1-positive and IFNAR1-deficient mice. Mx1 gene transcription was analyzed by real-time RT-PCR, 7 days after electroinjection of plasmid coding for MuIFN-α6T, MuIFN-λ3 or the empty vector (mock) in 6 week-old Mx1-positive mice (2 BALB.A2G-Mx1 and 2 B6.A2G-Mx1 mice, grouped as Mx1/WT mice) or in 8 week-old Mx1/IFNAR1-KO mice. Results are expressed as Mx1 cDNA copies per β-actin cDNA copy. Graphs present results for individual mice and the mean for each group, for one representative experiment.

We tested the efficacy of the procedure by following plasmid-driven expression of luciferase, using in vivo imaging. As shown in Figure 3, luciferase expression was readily detectable in the tibialis muscle after 2 days, and lasted up to 3 or 4 months after a single plasmid electroinjection. Then, to test whether IFN could be expressed in vivo, in this experimental setting, mice were electroinjected in the tibialis anterior muscle, with plasmid DNA coding for MuIFN-α6T (accession AY220465) or for a mutant of this IFN carrying a glycosylation site (D68N mutation). PCR analysis and sequencing of PCR products confirmed that the IFN subtype expressed in the muscle corresponded to the subtype expressed by the injected plasmid (data not shown). Two days and seven days after electroinjection, both glycosylated and non-glycosylated forms of circulating IFN-α6T, expressed from tibialis muscles, induced the expression of various ISGs (OASl2, Mx1, IRF7 and Ifit1) in the injected muscle but also in liver, spleen and kidney. ISGs were also upregulated, but to a lesser level, in the brain and in the spinal cord (Figures 4 and 5, Tables 4 and 5, and data not shown). When the empty vector was electroinjected, upregulation of ISG expression was detectable in the injected muscle but hardly, if at all, in other tissues. Experiments conducted in IFNAR1-KO mice failed to reveal transcriptional upregulation of ISGs by IFN-α6T (Figure 5, Tables 4 and 5), showing that the induction of ISGs, observed in mice carrying the type I IFN receptor gene, was indeed type-I IFN-dependent.

**Table 4:** Induction of OASl2 gene expression in response to circulating IFNs.

Expt.	Mice	Expressed IFN	n	Liver^a^	Spleen^a^	Kidney^a^	Brain^a^	Spinal cord^a^
1	FVB	IFN-α6T	5	32±7,5	33±5,9	38±4,1	50±19	-
		IFN-α6T/D78N	5	23±6,4	21±2,2	33±8	24±6	-
		IFN-λ	-	-	-	-	-	-
2	FVB	IFN-α6T	6	16±8,6	30±7,2	15±2	32±19	17±4,2
		IFN-α6T/D78N	5	16±10	28±10	26±14	27±10	20±6
		IFN-λ	6	<2	<2	6±0,9	2,5±0,6	<2
3	Mx1/WT b	IFN-α6T	3	9,2±3,3	-	14±3	-	-
		IFN-α6T/D78N	3	6,1 ±1,5	-	9,7±0,5	-	-
		IFN-λ	2	2,7±2,2	-	27±5,2	-	-
4	Mx1/WT c	IFN-α6T	4	2,3±1,7	-	10±2,4	39±18	-
		IFN-α6T/D78N	-	-	-	-	-	-
		IFN-λ	4	<2	-	11±1,4	3,1±0,7	-
5	Mx1/IFNAR1-KO	IFN-α6T	2	<2	-	<2	<2	-
		IFN-α6T/D78N	-	-	-	-	-	-
		IFN-λ	2	<2	-	15±4,6	2,5±0,3	-

a OASl2 induction (mean±SD) by IFNs:  = OASl2 expression determined by real-time RT-PCR in organs of mice electroinjected with the plasmid expressing the indicated IFN divided by OASl2 expression in the corresponding organ of mice electroinjected with the empty vector.

b BALB.A2G-Mx1 mice.

c BALB.A2G-Mx1 (n = 2) and B6.A2G-Mx1 (n = 2).

**Table 5:** Induction of Mx1 gene expression in response to circulating IFNs.

Expt.	Mice	Expressed IFN	n	Liver^a^	Kidney^a^	Brain^a^
3	Mx1/WT^b^	IFN-α6T	3	7±6	33±7	-
		IFN-α6T/D78N	3	11±3	21±5	-
		IFN-λ	2	3±2	23±5,8	-
4	Mx1/WT^c^	IFN-α6T	4	19±6	27±11	9,1±6,5
		IFN-α6T/D78N	-	-	-	-
		IFN-λ	4	2,8±1,2	8,4±2,4	2,3±0,4
5	Mx1/IFNAR1-KO	IFN-α6T	2	<2	<2	<2
		IFN-α6T/D78N	-	-	-	-
		IFN-λ	2	<2	12,5±4,5	<2

a Mx1 induction (mean±SD) by IFNs:  = Mx1 expression determined by real-time RT-PCR in organs of mice electroinjected with the plasmid expressing the indicated IFN divided by Mx1 expression in the corresponding organ of mice electroinjected with the empty vector.

b BALB.A2G-Mx1 mice.

c BALB.A2G-Mx1 (n = 2) and B6.A2G-Mx1 (n = 2).

Thus, electrotransfer of plasmid DNA in vivo allows the expression of circulating IFN which activates ISG expression in the tissues examined. In this experimental setting, no significant difference was detected between the activities of glycosylated and non-glycosylated forms of IFN-α6T.

### Response to systemic type III IFN

We used this in vivo expression strategy to compare the tissue specificities of the responses to type I and type III IFNs. Seven days after electrotransfer, we measured, by real-time RT-PCR, ISG expression in the organs of mice that received plasmids coding for either IFN-α6T or IFN-λ3 (Figures 4 and 5 and Tables 4 and 5). Interestingly, although IFN-α6T induced bona fide ISG expression in all organs tested, response to IFN-λ3 exhibited some tissue specificity. In response to IFN-λ3, expression of OASl2 (Figure 4), Mx1 (Figure 5), and IFIT1 (not shown) was close to background in the brain, spinal cord, spleen, liver, and muscle but was detected in the kidney. In different experimental settings (Table 4), induction of OASl2 expression by IFN-λ3 was ≤3.1±0.7 in the brain but ranged from 6±0.9 to 27±5.2 in the kidney. Accordingly, induction of Mx1 expression in mice carrying functional *Mx1* alleles was ≤2.3±0.4 in the brain but ranged from 8.4±2.4 to 29±0.6 in the kidney. Experiments performed in IFNAR1-KO mice (Figure 5 and Table 5) indicated that the induction of ISG expression observed with IFN-λ3 did not depend on the activation of the type I IFN system.

### Tissue specificity of IL-28Rα expression

**Figure 6:**
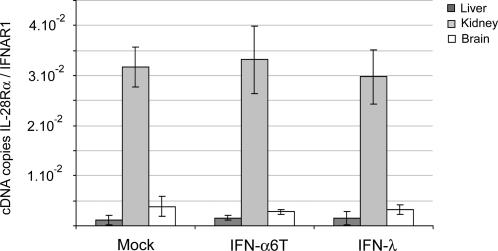
IL-28Rα expression exhibits tissue specificity. IL-28Rα and IFNAR1 expressions were determined 7 days after electroinjection of plasmids coding for MuIFN-λ3 (right) or MuIFN-α6T (center), or after electroinjection of an empty vector (left), in 6 week-old BALB.A2G-Mx1 mice. IL-28Rα and IFNAR1 expressions were measured by real-time RT-PCR. Results are expressed as means and SD of the ratio between IL28Rα and IFNAR1 cDNA copies.

In cell lines, the response to type III IFN was shown to be related to the expression level of the α subunit of the IL-28 receptor. Differential expression of IL-28Rα could thus explain the tissue selectivity of IFN-λ responses in vivo. We used real-time RT-PCR to compare the expression levels of IL-28Rα and of the ubiquitously expressed IFNAR1 subunit of the type I IFN receptor, in the brain, liver and kidney. Expression of IFNAR1 and IL-28Rα were influenced neither by IFN-α nor by IFN-λ expression (Figure 6). In the kidney, which showed good responsiveness to type III IFN, IL-28Rα expression was clearly higher than in brain and liver (Figure 6).

### IFN-λ induces ISG expression in epithelial cells but not in endothelial cells

In order to identify the cells responding to type I and type III IFNs in vivo, we performed immunohistofluorescence using Mx1 as a marker of the IFN response. On one hand, we studied the IFN response in the kidney which was found to respond readily to both IFN-α and IFN-λ (see Figure 4). On the other hand, we examined the IFN response in the brain. In contrast to the kidney, this organ readily responded to type I IFN but hardly responded to type III IFN.

**Figure 7:**
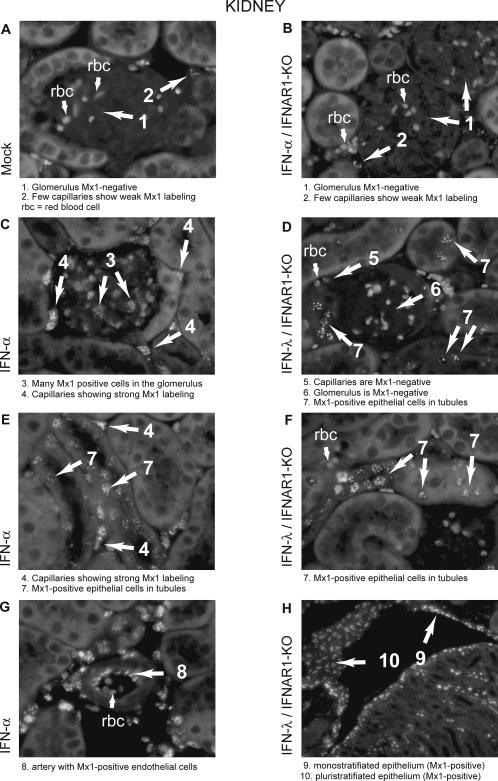
IFN-α and IFN-λ responding cells in the kidney. Mx1 expression, detected by immunohistochemistry (white nuclear spots), 7 days after electroinjection of a plasmid coding for MuIFN-α6T or MuIFN-λ3. Sections of the kidney from: A. control Mx1/WT mouse electroinjected with the empty vector. Note that few cells (mostly endothelial cells) were weakly Mx1-positive. B. control Mx1/IFNAR1-KO mouse electroinjected with a plasmid coding for IFN-α6T. C–E–G: Mx1/WT mouse electroinjected with a plasmid coding for IFN-α6T. D–F–H: Mx1/IFNAR1-KO mouse electroinjected with a plasmid coding for IFN-λ3.

**Figure 8:**
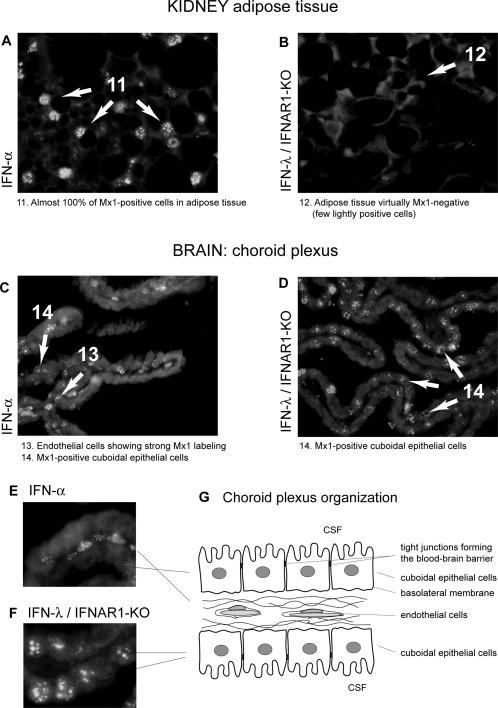
IFN-α and IFN-λ responding cells in the kidney adipose tissue and in the brain. Mx1 expression, detected by immunohistochemistry (as white nuclear spots), 7 days after electroinjection of a plasmid coding for MuIFN-α6T or MuIFN-λ3. A–C–E: Mx1/WT mouse electroinjected with a plasmid coding for IFN-α6T. B–D–F: Mx1/IFNAR1-KO mouse electroinjected with a plasmid coding for IFN-λ3. A–B: sections showing the kidney adipose tissue. C–D: brain sections showing the choroid plexus of the 4th ventricle. E–F: Higher magnification of the choroid plexus. G: Cartoon showing the structural organization of the choroid plexus.

In the kidney, IFN-α-induced Mx1 expression was widespread (Figures 7C, 7E, 7G, and 8A). Mx1 labeling was prominent in endothelial cells (Figures 7C, 7E, 7G) but Mx1-positive cells also included epithelial cells of the tubules and of the urinary epithelium (not shown). The neighboring adipose tissue was also strongly responsive to IFN-α6T (Figure 8A). In contrast, Mx1 expression in response to IFN-λ was strikingly restricted to epithelial cells (Figures 7D, 7F). Labeling of the urinary epithelium was prominent (Figure 7H) (much stronger than in response to IFN-α expression). Glomeruli were negative (Figure 7D). In the cortex and medulla, only epithelial cells were positive. Adipose tissue showed background-like labeling (Figure 8B).

In the brain, very few cells responded to IFN-λ, as expected from the very low expression of ISGs in this organ. These cells appeared to correspond to rare epithelial cells of the meninges and to cells of the choroid plexus. In the choroid plexus, the comparison between Mx1 expression in response to IFN-α and to IFN-λ was exemplar (Figure 8C–F). IFN-α induced mostly Mx1 expression in the endothelial cells of the vessels comprised between the two monolayers of cuboidal epithelial cells (Figure 8C and 8E). Some epithelial cells were also Mx1-positive. In response to IFN-λ, Mx1 expression was prominent in epithelial cells but absent from endothelial cells (Figure 8D and 8F).

**Figure 9:**
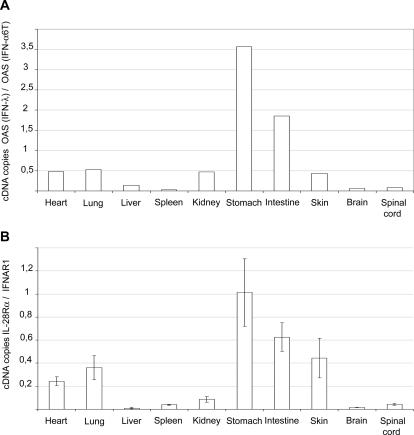
Correlation between the relative responsiveness of organs to IFN-λ and the relative expression of IL-28Rα and IFNAR1. A. Relative functional influence of IFN-λ over IFN-α in various organs. The histogram shows, for each organ, the ratio between OASl2 expression in response to circulating IFN-λ and OASl2 expression in response to circulating IFN-α (mean of two mice), 7 days after electrotransfer of the expression plasmids. B. Relative expression, measured by real-time RT-PCR, of IL-28Rα and IFNAR1 (mean and SD from 5 mice).

In view of the striking restriction of the IFN-λ response to epithelial cells in the brain and in the kidney, we tested whether the responsiveness of different tissues to IFN-λ would parallel their epithelial nature. Thus, we used real-time RT-PCR to compare, in different tissues, i) ISG induction in response to systemically expressed IFN-λ versus IFN-α (Figure 9A), and ii) IL-28Rα versus IFNAR1 expression (Figure 9B). Response of the tissues to IFN-λ (over IFN-α) nicely paralleled IL-28Rα (over IFNAR1) expression. Interestingly, tissues like stomach, intestine, skin, and lung, which have an important epithelium component showed the highest IFN-λ over IFN-α responsiveness. The small apparent differences seen between relative expressions of IL-28Rα (over that of IFNAR1) in tissues of gastro-intestinal tract and in lungs or skin were not significant. Also, these differences did not appear when considering IL-28Rα expression alone (data not shown). In contrast, nervous tissues and spleen responded very poorly to IFN-λ and expressed small amounts of IL-28Rα. Surprisingly, the liver responded poorly to IFN-λ and expressed low amounts of IL-28Rα, in spite of the epithelial nature of the hepatocytes. In contrast, the response of the heart was surprisingly high.

## Discussion

### Tissue specificity of IFN-λ expression

Many data converge to show that type I IFN can be expressed by virtually all nucleated cells, including some neurons. In contrast, little is known about the specificity of IFN-λ expression. Upregulation of IFN-λ transcription has been shown to depend on the same stimuli, sensors, and signal transduction pathways as those involved in type I IFN production [17]–[21],[28]. IFN-λ expression has been mainly described in vitro, in MD-DCs, pDCs, macrophages, and in numerous lymphoid, myeloid and epithelial cancer cell lines [18],[28]. In these studies, IFN-α/β and IFN-λ have been shown to be expressed simultaneously. In MD-DCs and in pDCs, upon influenza A or Sendai virus infection, IFN-α/β and IFN-λ were expressed at the same order of magnitude and with similar kinetics [43].

Our data show that expression of IFN-λ in the central nervous system is minimal, even under conditions of strong IFN-α and IFN-β expression, as those observed after infection by LACVdelNSs or TMEV-GDVII. In contrast, in the liver, IFN-λ was readily expressed after both LDV and MHV-A59 infections. The difference between relative type III and type I IFN expression levels detected in the liver and in the brain was highly significant in the case of C57BL/6 mice infected i.p. with LDV or infected i.c. with MHV-A59. A similar trend of low relative expression of IFN-λ in the brain was observed with the other infection models (different viruses and different mouse strains). However, our study does not exclude a possible influence of the mouse genetic background in the relative expression of type I and type III IFN genes.

Nevertheless, our results show that some differential tissue specificity exists in the production of type I and type III IFNs. This suggests that the molecular pathways leading to type I and type III IFN gene expression vary either qualitatively (some specific factors required for IFN-λ gene induction) or quantitatively (different thresholds of sensors, signal transduction or transcription factors required for the activation of type I and type III IFNs). The tissue specificity of IFN-λ production observed in this work probably results from cell type specificity. In vitro, IFN-λ was shown to be notably produced by MD-DCs and pDCs [43]. If these cells are also important IFN-λ producers in vivo, the paucity of DCs, in particular of pDCs, in the CNS might be the reason for the low expression of type III IFN in this organ.

### Tissue and cell specificity of the IFN-λ response

In cell lines, IFN-λ responses have been shown to correlate with expression of IL-28Rα. On the basis of IL-28Rα expression and of IFN-λ responsiveness of cell lines and primary cells, it was suggested that IFN-λ could be primarily expressed by cells of epithelial origin. Accordingly, in vivo, IFN-λ proved to be effective against some viruses known to infect epithelial cells such as Herpes simplex virus-2 [17]. Indirect evidence also comes from the fact that Yaba-like disease virus, a virus with tropism for the dermis was found to produce a type III IFN antagonist protein [11]. However, until now, no direct in vivo data identified the cells responding to IFN-λ.

Here, we show, by immunohistochemistry, that the response to IFN-λ involves primarily epithelial cells, at least in the kidney and in the CNS. In the kidney, Mx1 expression in response to IFN-λ was notably prominent in cells of the pluristratifiated urinary epithelium. In contrast, endothelial cells which responded nicely to IFN-α failed to respond to IFN-λ. In the choroid plexus of the brain, response to IFN-α was most prominent in endothelial cells and detectable in cuboidal epithelial cells. In contrast, response to IFN-λ was only detectable in cuboidal epithelial cells. At the tissue level, responsiveness to IFN-λ, as measured by ISG induction, correlated with IL-28Rα over IFNAR1 expression. Again, epithelium-rich tissues such as stomach, intestine, skin or lung were responsive to IFN-λ. It is not clear, however, why the liver was not more responsive and why the heart appeared to be as responsive as the lung.

IFN-λ was reported to share, with type I IFN, immunomodulatory activities. For instance, IFN-λ was found to modulate the Th1/Th2 balance of the immune responses [32]. However, in agreement with previous studies, our data show that neither endothelial cells nor spleen cells, two important components of homing and activation of immune cells, responded detectably to IFN-λ, though the response of a small cell population could easily have been undetected. It will be of interest to identify the target cells that mediate the immunomodulatory function of IFN-λ.

### Lack of IFN-λ response in the central nervous system

Type I IFN turned out to have much impact on CNS pathologies. On one hand, type I IFN was shown to be instrumental in the resistance of humans and mice to neurotropic viral infections [44],[45]. On the other hand, type I IFN proved to be beneficial against autoimmune disorders like multiple sclerosis [46],[47] and the murine experimental autoimmune encephalitis [48]. IFN-β was shown to decrease the relapse rate and disease activity in relapsing-remitting MS [49]. However, exposure to type I IFN can also cause adverse effects. IFN treatment often triggers flu-like symptoms. When prolonged, for instance in the case of hepatitis C treatment, type I IFN treatment can lead to neurological or neuropsychiatric adverse effects like depression [50],[51].

IFN-λ could represent an interesting alternative to type I IFN. Indeed, IFN-λ appears to activate the same set of genes as type I IFN and most biological functions of type I IFN appear to be shared by type III IFN. We observed that the CNS is both a poor IFN-λ producer and a poor responder to this cytokine. In the CNS, the blood-brain barrier is mostly made of the tight junctions that bridge the endothelial cells and thus prevent the diffusion of metabolites from the blood to the CNS parenchyma. The lack of responsiveness of endothelial cells to circulating IFN-λ could thus explain the global absence of response to IFN-λ in the CNS. In the choroid plexus, however, endothelial cells are fenestrated. In this structure, the blood-brain barrier is formed by the tight junctions occurring between the cuboidal epithelial cells (Figure 8G). Response of these cells to IFN-λ suggests that they express the IFN-λ receptor on their basolateral membrane which is accessible to factors diffusing from the bloodstream. The low responsiveness of the CNS to IFN-λ does not appear to result solely from the combination of the blood-brain barrier and lack of endothelial cell responsiveness. Our RT-PCR data show that expression of the IL-28Rα receptor chain is very low in the entire brain. This suggests that, even in inflammatory conditions (such as in MS or during viral infection) known to affect the integrity of the blood-brain barrier, the CNS would be expected to respond poorly to IFN-λ. This fits with the observation that IFNAR1-KO mice (which have an intact IFN-λ system) exhibit extreme susceptibility to many neurotropic viral infections [45]. It will be of interest to test whether, owing to the low responsiveness of the CNS, IFN-λ would exhibit less toxicity than IFN-α/β. This might be of interest if the effective targets of the IFN treatment are in the periphery and, of course, responsive to IFN-λ.

### Redundancy of type I and type III IFN systems and relevance to viral pathogenesis

Although type I and type III IFNs signal through different receptors, these two IFN families share common features. Production of both IFN types can be triggered by the same stimuli and responses of cells to type I and type III IFNs involves the upregulation of the same set of genes. Why these two seemingly redundant systems co-evolved is not fully clear. Previous data based on cell lines and primary cells responsiveness to IFN-λ suggested that a key difference between the type I and type III IFN systems could be the cell specificity of IFN-λ receptor expression [18],[26]. Our work confirms that, in vivo, a major difference between the type I and type III IFN systems is the cell type-restricted nature of responses to IFN-λ. Type III IFN appears to have evolved primarily as a protection of epithelial cells. However, type I IFN also acts on these cells, leaving open the question of redundancy.

On one hand, IFN-λ could be viewed as a leftover of an ancestral antiviral protection system that arose to protect simple organisms. In the evolution, type III IFN-like genes, which occur in the genome of the fish, appear to have preceded type I IFN genes that emerged with the development of birds and tetrapods [52]. Type I IFN would have evolved faster to become the primary antiviral protection system, active in many cell types. In this hypothesis, IFN-λ would only play the role of a back-up system.

On the other hand, the co-existence of two systems with overlapping specificities might have been selected because both systems contribute to the protection against live-threatening and/or widespread pathogenic viruses or microorganisms. Our data suggest that the primary function of IFN-λ would be the protection of epithelial structures. Many viruses use epithelial cells as primary replication sites. These include viruses like poxviruses, herpesviruses and influenza virus that could have had enough impact on species populations to drive some evolution of the genomes. The effect of IFN-λ against vaginal infection by HSV-2 [17], the inverse correlation between rhinovirus-induced IFN-λ expression and viral load in infected volunteers [53], and the antagonistic activity of Yaba-like virus against IFN-λ [11], support an active role for this IFN. IFN-λ is thus also expected to contribute to the defense of respiratory epithelia, against influenza virus. Accordingly, recent findings suggest that IFN-λ contributes to the protection of airways against influenza A virus, through induction of Mx gene expression (Markus Mordstein and Peter Staeheli, unpublished observations). IFN-λ might also be instrumental in the early defense of the intestinal mucosa against very common pathogens such as rotaviruses or possibly against bacteria. Further studies are needed to confirm that, in these tissues, the primary targets of IFN-λ activity are also the epithelial cells, and to evaluate how much protection is added by the IFN-λ system to the very potent IFN-α/β system. The notion that some differential regulation exists in the production of type I and type III IFNs might also broaden the range of the response or accelerate the reactivity of the body to some specific pathogens.

## Materials and Methods

### Mice

3–4 week-old female FVB/N, 129/Sv, C57BL/6 mice (infection experiments) and 7–8 week-old female or male FVB/N mice (electroinjection experiments) were obtained from Charles River Laboratories or from the animal facility of the Univ. of Louvain, Belgium. Congenic mice carrying a functional Mx1 gene were from the breeding colony of the Univ. of Freiburg, Germany. These mice were BALB.A2G-Mx1 and B6.A2G-Mx1 (designated Mx1/WT) [54] as well as B6.A2G-Mx1 mice lacking a functional type I IFN receptor (designated Mx1/IFNAR1-KO) [55]. Handling of mice and experimental procedures were conducted in accordance with national and institutional guidelines for animal care and use (Agreement ref. UCL/MD/2006/034).

### Viruses and infection

Viruses used in this study were: Theiler's murine encephalomyelitis virus (TMEV) persistent strain DA (DA1 molecular clone), and neurovirulent strain GDVII [40], La Crosse virus deleted from the NSs gene (LACVdelNSs) [56], mouse hepatitis virus, strain A59 (MHV-A59) [57] and lactate dehydrogenase-elevating virus of the Riley strain (LDV) [58].

Intracerebral infections (i.c.) were done by injection of 40 microliters of serum-free medium containing 10^3^ PFU of TMEV(GDVII), 10^5^ PFU of TMEV(DA), or 2×10^4^ TCID_50_ of MHV-A59. Control mice were injected with 40 microliters of serum-free culture medium. Intraperitoneal (i.p.) infections were performed by injection of 250 microliters of serum-free medium containing 10^4^ PFU of LACVdelNSs, 2×10^7^ ID_50_ of LDV, or 1 or 2×10^4^ TCID_50_ of MHV-A59.

### RNA extraction and real-time reverse transcription (RT)-PCR

Mice were anesthetized and perfused with PBS before organs harvest. RNA was isolated from organs using the technique described by Chomczynski and Sacchi [59] and reverse-transcribed as previously described [60]. Real-time RT-PCR was performed, as described previously [60], using SybrGreen and the iCycler or the MyIQ™ apparatus (Biorad). Standards consisted of 10-fold dilutions of known concentrations of murine genomic DNA, of plasmids carrying the PCR fragment of interest (pCR4-Topo, Invitrogen) or plasmid pcDNA3-IFN-α5 [10] or pEF-IFN-λ3 [16] (kindly provided by S. Kotenko). Primers sequences and PCR conditions used are presented in Table 1. The IFN-subtype specificity of primers for IFN-α5 was confirmed. No PCR product was detected when plasmids encoding the other IFN-α subtypes were used as templates. Moreover, when genomic DNA was used as template, the IFN-α5 gene segment was specifically amplified, as confirmed by sequencing of the PCR products.

### Plasmids

The firefly luciferase gene was cloned from pGL3 (Promega) in pcDNA3 (Invitrogen) using *Hin*dIII-*Xba*I restriction sites, to yield pCS41. Plasmid pcDNA3-muIFNα6T [10] was subjected to site directed mutagenesis [61] with oligonucleotide TM439 (5′ GGA GGG TTG CAT **T**CC AAG CAG CAG A 3′) to generate the Asp to Asn78 mutant (D78N) that carries a N-glycosylation site. The mutated IFN-α6T region was recloned in pcDNA3 and sequenced to make sure that no unexpected mutation occurred during the mutagenesis procedure.

MuIFN-λ3, was cloned from pEF-2-mIFN-λ3 [16] into pcDNA3 (Invitrogen) using *Asp*718-*Eco*RI restriction sites. The human IFNGR2 signal sequence and the N-terminal FLAG coding sequences present in pEF-2-mIFN-λ3 were replaced by a sequence encoding the wild-type murine IFN-λ3 signal sequence. To this end, the 3′ complementary primers TM723 (5′ AAA GGT ACC GCC ACC ATG CTC CTC CTG CTG TTG CCT CTG CTG CTG GCC GCA 3′) and TM724 (5′ AAA GGA TCC GCT TGG GTT CTT GCT AGC ACT GCG GCC AGC AGC AGA GGC AA 3′) were used for PCR and the resulting fragment was cloned in the recombinant plasmid using the *Asp*718 and *Bam*HI restriction sites. The muIFN-λ3 region was sequenced to make sure that no unexpected mutation occurred during PCR and subcloning steps. The plasmid obtained, pcDNA3-IFN-λ3, encodes a wild-type muIFN-λ3 with a wild-type signal sequence. A similar procedure was followed to obtain pcDNA3-IFN-λ2 from pEF-2-mIFN-λ2 [16].

### DNA electroinjection

Mice were anesthetized with 200 µl of a mix of Medetomidin hydrochlorid 100 µg/ml (Domitor) and Ketamine 500 µg/ml (Anesketin) given i.m. Before DNA injection, mice were shaved locally, using depilatory cream. 10 µg of endotoxin free plasmid DNA (Qiagen endofree) in 25 µl of PBS were injected in the left and right tibialis anterior muscles of the mice. Electric pulses (80 V per 4 mm, 8 pulses, 20 msec/pulse, pause: 480 msec) were then administered using a Cliniporator system (Cliniporator, IGEA, Carpi, Italy) equipped with 4 mm electrode plates [62]. For all experiments, conductive gel was used to ensure electrical contact with the skin (EKO-GEL, ultrasound transmission gel, Egna, Italy). Mice were then woken up by i.m. injection of 250 µl of Atipamezol 500 µg/ml (Antisedan).

### In vivo imaging

Mice were anesthetized as for DNA electroinjection and given 3 mg of Luciferin (Xenogen) in 100 µl of PBS, intraperitoneally. 10 min after luciferin injection, luciferase activity was monitored in vivo using a CCD camera (IVIS 50, Xenogen) [63]. Mice were then woken up, as described above.

### Immunohistochemistry

Mice were anesthetized before being euthanized for organs harvest. They were perfused with PBS. Freshly collected brains and kidneys were immersed in buffered formaldehyde 4% for 24h at room temperature and then embedded in paraffin. Tissue sections of 8 µm in thickness were cut, placed on SuperFrost Plus slides, dried at 37°C overnight, and processed by standard methods for immunohistochemistry. Briefly, sections were deparaffinized, permeabilized for 5 min in phosphate-buffered saline (PBS) containing 0.1% Triton X-100, and washed in PBS. Sections were then treated for 90 min at 97°C in sodium citrate buffer 0.01 M - pH 5.8, to unmask antigens. Blocking was performed by incubating sections for 1 hour with normal goat Serum (Sigma) diluted 1/50 in PBS. Then, immunolabeling was done in blocking solution containing the antibodies. Mx1 protein was detected with rabbit polyclonal antibody AP5 [64] that recognizes the C-terminal 16 amino acids of Mx1. It was used at a dilution of 1/150. For immunofluorescent labeling, the secondary antibody (at 1/800) was a goat anti-rabbit antibody coupled to Alexa 488 (Molecular Probes).
